# Interobserver Reliability of Endoscopic Ultrasonography: Literature Review

**DOI:** 10.3390/diagnostics10110953

**Published:** 2020-11-15

**Authors:** Akira Yamamiya, Atsushi Irisawa, Ken Kashima, Yasuhito Kunogi, Kazunori Nagashima, Takahito Minaguchi, Naoya Izawa, Akane Yamabe, Koki Hoshi, Keiichi Tominaga, Makoto Iijima, Kenichi Goda

**Affiliations:** Department of Gastroenterology, Dokkyo Medical University School of Medicine, 880 Kitakobayashi Mibu, Tochigi 321-0293, Japan; akira-y@dokkyomed.ac.jp (A.Y.); ken-k@dokkyomed.ac.jp (K.K.); ykunogi@dokkyomed.ac.jp (Y.K.); n-kazu@dokkyomed.ac.jp (K.N.); takahito@dokkyomed.ac.jp (T.M.); izawanao@dokkyomed.ac.jp (N.I.); yamabe@dokkyomed.ac.jp (A.Y.); hoshi@dokkyomed.ac.jp (K.H.); tominaga@dokkyomed.ac.jp (K.T.); mkiijima@dokkyomed.ac.jp (M.I.); goda@dokkyomed.ac.jp (K.G.)

**Keywords:** chronic pancreatitis, endoscopic ultrasonography, gallbladder wall thickening, interobserver reliability, intrapancreatic accessory spleen, pancreatic cystic lesion, lymphadenopathy, solid pancreatic lesion, subepithelial lesion

## Abstract

Endoscopic ultrasonography (EUS) has been applied to the diagnosis of various digestive disorders. Although it has been widely accepted and its diagnostic value is high, the dependence of EUS diagnosis on image interpretation done by the endosonographer has persisted as an important difficulty. Consequently, high interobserver reliability (IOR) in EUS diagnosis is important to demonstrate the reliability of EUS diagnosis. We reviewed the literature on the IOR of EUS diagnosis for various diseases such as chronic pancreatitis, pancreatic solid/cystic mass, lymphadenopathy, and gastrointestinal and subepithelial lesions. The IOR of EUS diagnosis differs depending on the disease; moreover, EUS findings with high IOR and those with IOR that was not necessarily high were used as diagnostic criteria. Therefore, to further increase the value of EUS diagnosis, EUS diagnostic criteria with high diagnostic characteristics based on EUS findings with high IOR must be established.

## 1. Introduction

Endoscopic ultrasonography (EUS) was first reported in 1980 [1,2]. Since then, EUS has been applied to the diagnosis and treatment of pancreatic diseases [3,4,5,6,7]. Currently, EUS is used to diagnose diseases of various organs, including the pancreas. Generally, EUS is regarded as an effective modality because of its excellent image resolution, which allows the detection of small lesions and accurate assessment of staging in addition to the possibility of tissue sampling. Consequently, numerous reports of the diagnostic ability of EUS exist. Despite its benefits, EUS is not always perfect; limitations that hinder its accuracy are reportedly attributed to the following: (i) EUS imaging and diagnosis depend on the skill of the physicians, i.e., inter-operator variation; (ii) endoscopic ultrasound-guided fine needle aspiration (EUS-FNA) is often necessary for differential diagnosis because distinguishing between neoplastic and non-neoplastic tissue is difficult with EUS imaging; and (iii) conditions such as biliary/pancreatic duct stenting and chronic pancreatitis can interfere with the detection and staging of small lesions [8,9,10,11,12,13]. Particularly, inter-operator variation presents major difficulties. Consequently, high inter-observer reliability (IOR) is important to improve the reliability of imaging by EUS.

Currently, numerous diagnostic criteria based on EUS for various diseases are available. In these diagnostic criteria, a high IOR of EUS diagnostic findings is crucially important to support its diagnostic value. Some studies of IOR of EUS diagnosis have been conducted for various gastrointestinal diseases. This review presents a summary of the literature regarding the IOR of EUS diagnosis and explains some of the “wonders and problems” of current EUS diagnosis. For this study, IOR is expressed as kappa (*K*) values, which are defined as follows: <0, no agreement; 0–0.20, slight; 0.21–0.40, fair; 0.41–0.60, moderate; 0.61–0.80, high; and 0.81–1.00, almost perfect [14]. The literature from 1966 through 2019 was searched in PubMed using the keywords EUS and IOR.

## 2. Chronic Pancreatitis

EUS has been employed for the diagnosis of patients with chronic pancreatitis (CP) or suspected CP for over 20 years. Results of an earlier study suggested that CP was detectable by EUS even when findings of noninvasive imaging studies, such as computed tomography and magnetic resonance imaging, were negative [15]. Lees et al. demonstrated the usefulness of EUS morphology and histology after pancreatic resection in post-pancreatic resection specimens [16]. The results showed that CP was diagnosed histologically in six of the seven patients with CP findings on EUS. Other reports have described good correlation between EUS findings of CP and histological findings of CP, except in elderly patients [17,18]. Albashir et al. reported that the diagnostic performance of EUS based on histological findings was 84% for sensitivity and 100% for specificity [19].

Because of the well-known utility of EUS in the diagnosis of CP, this has been based on standard scoring of CP, defined by the International Working Group as standard criteria (SC) since these guidelines were published in 1998 [20] (Supplementary Table S1). The nine findings adopted were the following: hyperechoic foci, hyperechoic strands, lobularity, calcifications/stones, cysts, main pancreatic duct dilation, main pancreatic duct irregularity, hyperechoic main pancreatic duct (MPD) walls, and visible side branches. Subsequently, new diagnostic criteria for CP, i.e., the Rosemont Criteria (RC), were adopted in 2009 [21] (Supplementary Table S2).

The IOR of each finding has been reported in numerous studies (Table 1). The first report on IOR was by Wallence et al. in 2001 [22]. The *K* value of duct dilatation was 0.61 (high). That of lobularity was 0.51 (moderate), whereas that of the other features was <0.4 (slight and fair). Stevens et al. reported a similar result. Only the IOR of duct dilation was high (*K* = 0.61) [23]. Del Pozo et al. reported that the *K* values of lobularity, cysts, and MPD dilation were large (0.75, 0.66, and 075, respectively), whereas the IOR was only fair in lobularity with honeycombing (*K* = 0.31) [24]. Gardner et al. reported that the IOR is almost perfect in cysts (*K* = 1.00) and high in strands (*K* = 0.62) [25]; the other findings had low concordance rates: moderate in duct dilation, hyperechoic MPD wall, and lobularity (*K* = 0.53, 0.53, and 0.44, respectively) and fair in hyperechoic foci (*K* = 0.39). Lieb et al. reported that the IOR of stones and duct dilation were high (*K* = 0.78 and 0.77, respectively), whereas those of hyperechoic foci, strands, and side branch dilation were slight (*K* = 0.19, 0.07, and 0.11, respectively) [26]. A T study by Topazian et al. found low IOR in all cases: the IOR of cysts, which was the highest value (*K* = 0.48), was fair; those of the other findings were slight [27].

Final diagnosis of CP by EUS based on SC and RC has also been reported. The assessment of IOR of SC has been studied previously. It was found to be moderate at best. Wallence et al., Stevens et al., Del Pozo et al., and Kalmin et al. reported moderate IORs of the final diagnoses (*K* = 0.45, 0.54, 0.53, 0.50, respectively) [22,23,24,28]. The IOR of the final diagnosis in the study reported by Lieb et al. was fair (*K* = 0.39) [26].

Furthermore, several studies evaluated the IOR of the final diagnosis for both SC and RC. Del Pozo et al. reported that the IOR of the final diagnosis was moderate for both criteria (*K* = 0.53 for SC and *K* = 0.46 for RC) [24]. The study reported by Stevens et al. revealed the IOR of the final diagnosis as moderate for SC (*K* = 0.54) and high for RC (*K* = 0.65). However, the difference was not statistically significant (*p* = 0.12) [23]. Kalmin et al. reported that the IOR of the final diagnosis was moderate for SC (*K* = 0.50) and fair for RC (*K* = 0.27) [28]. These results suggest that the IOR did not seem to improve with the RC compared with the SC.

The examiners participating in most of the studies were experts. However, some studies have investigated differences in IOR according to years of experience with EUS. Wallence et al. reported the IOR of the final diagnosis as moderate for fellows and experts (*K* = 0.42 and 0.46, respectively) [22]. Stevens et al. reported no significant difference in IOR based on years of experience, irrespective of the criteria used (i.e., SC or RC) [16].

Although the IOR of the respective findings for CP varied, several reports have described that the IOR of the final diagnosis produced using both SC and RC is moderate. Moreover, the findings suggest that the IOR might not depend on the years of experience with EUS.

## 3. Solid Pancreatic Lesion

Imaging of a solid pancreatic lesion (SPL) is fundamentally important for treatment. SPLs include pancreatic adenocarcinoma, inflammatory mass in the context of CP, pancreatic neuroendocrine tumor, autoimmune pancreatitis, and other tumors. Among them, pancreatic adenocarcinoma has a particularly poor prognosis. For that reason, early detection is desirable. EUS plays a major role as a diagnostic modality: even small lesions of 5 mm can be visualized reliably by EUS. Moreover, contrast-enhanced harmonic endoscopic ultrasonography (CH-EUS) is an extremely useful test in the differentiation of SPL [29,30]. CH-EUS can assess tissue perfusion in real time without Doppler-related artifacts [30,31,32]. A recent study demonstrated that the microvascular pattern of SPL based on CH-EUS correlates closely with histological features [32,33]. Gong et al. reported that the sensitivity of CH-EUS for the differential diagnosis of pancreatic adenocarcinoma is 94%; the specificity was 89% [34].

Based on EUS, pancreatic adenocarcinomas are unevenly margined and well-defined. Small tumors present with a homogeneous hypoechoic appearance. However, as the tumor enlarges, a heterogeneous hyperechoic region develops in the center. Pancreatic duct dilation caudal to the mass and dilated surrounding branches accompanied by storage cysts are important indirect findings. CH-EUS often shows hypo-enhancement in the arteriovenous phase because of a desmoplastic reaction and an internal heterogeneous contrast pattern. Moreover, EUS images of neuroendocrine tumors (NETs) show a hypoechoic appearance with smooth edges, well-defined borders, and uniform interiors. In CH-EUS, it is of hyper-vascular-type. Inflammatory masses in the context of CP present an overall hypoechoic appearance. The boundary of the pancreatic parenchyma is somewhat obscured. They also often present with duct-penetrating signs.

Several studies have examined IOR of the diagnosis of SPL using CH-EUS (Table 2). Some studies have examined differences in IOR by years of experience with EUS. Kitano et al. reported that the combination of CH-EUS and EUS-FNA increased the sensitivity for the diagnosis of pancreatic adenocarcinoma to 100% [35]. After they evaluated the IOR of CH-EUS in 277 SPLs conducted by two experienced endo-sonographers, they reported a *K* value of 0.94 (almost perfect). Fusaroli et al. reported that the IOR of contrast uptake was moderate for all endo-sonographers (*K* = 0.56). Similar results were reported for experienced endo-sonographers (*K* = 0.56), and for non-experienced endo-sonographers (*K* = 0.55) [36]. Gincul et al. evaluated the IOR of CH-EUS by experienced endo-sonographers and non-experienced endo-sonographers. The overall IOR (*K* = 0.66) and the IOR of experienced (*K* = 0.65) and non-experienced (*K* = 0.76) endo-sonographers were high [37]. In this study, CH-EUS and EUS-FNA had high accuracy, with sensitivity of 95% and 96%, of 95% and 93% (*p* < 0.05). Bunganič et al. reported that the IOR of pancreatic adenocarcinoma diagnosis by EUS and CH-EUS was moderate (*K* = 0.45 and 0.60, respectively) [38]. The sensitivity and specificity of EUS and CH-EUS for pancreatic adenocarcinoma diagnosis were, respectively, 83.1% and 62.5% and 94.5% and 61.7%.

Palazzo et al. examined the IORs of the diagnoses of 81 cases of pancreatic NET (P-NET) [39]. The sensitivity and specificity of CH-EUS for the P-NET diagnosis were 96% and 82%, respectively. The IOR was high (*K* = 0.66) for all endo-sonographers, almost perfect (*K* = 0.83) for experienced endo-sonographers, and almost perfect (*K* = 0.82) for junior endo-sonographers.

Soares et al. evaluated the diagnostic ability of EUS-elastography (EUS-E) for SPLs [40]. Eleven endo-sonographers were divided into four groups: group A (long experience in EUS and EUS-E), group B (short experience in EUS and EUS-E), group C (long experience in EUS but no experience in EUS-E), and group D (no experience with EUS or EUS-E). The overall IOR was moderate (*K* = 0.42). The IOR of group A (*K* = 0.80) was significantly higher than that of groups B (*K* = 0.54), C (*K* = 0.54), and D (*K* = 0.28).

The IOR of SPL diagnosis by EUS was high. However, it might vary depending on the years of experience of endo-sonographers.

## 4. Pancreatic Cystic Lesion

Pancreatic cystic lesions include intraductal papillary mucinous neoplasm (IPMN), mucinous cystic neoplasm, and serous cystic neoplasm; all have a malignant potential [41,42,43]. EUS can provide morphological details of pancreatic cysts. However, a benign or malignant cyst is sometimes difficult to diagnose [44,45]. Earlier studies showed the diagnostic accuracy of EUS for pancreatic cystic lesions as 82–96% [46,47,48].

The first report of the IOR of pancreatic cystic lesion diagnosis was presented by Ahmad et al. in 2003 [49] (Table 3). Thirty-one cases with histologically classified pancreatic cystic lesions were reviewed independently by eight expert endo-sonographers. The IOR of diagnosing a benign or malignant cyst was fair (*K* = 0.24). Moreover, the IORs of the respective diagnostic finding were as follows: presence or absence of solid component, *K* = 0.43 (moderate); presence or absence of abnormal pancreatic duct, *K* = 0.29 (fair); debris, *K* = 0.21 (fair); septations, *K* = 0.30 (fair); presence or absence of margins, *K* = 0.01 (slight); and presence or absence of abnormal pancreatic parenchyma, *K* = 0.01 (slight). Fusaroli examined the IOR of pancreatic cystic lesion diagnosis by CH-EUS and found the IOR of contrast uptake to be moderate (*K* = 0.58) [36]. Gonzalez et al. examined the IOR of branch duct IPMN diagnosis by EUS and magnetic resonance cholangiopancreatography [50]. The IOR was moderate for PD = 5–9 mm (*K* = 0.45) and for abrupt change in PD (*K* = 0.529), fair for wall thickening (*K* = 0.259), and slight for non-enhanced mural nodule (*K* < 0) and lymphadenopathy (*K* < 0). In high-risk stigmata, the IOR was reported as slight for enhanced solid component (*K* = 0.12) and PD > 10 mm (*K* < 0). Jong et al. examined the IOR in three groups based on EUS experience [51]. The IOR of nodules was high in the expert group (*K* = 0.65) and fair in semi-expert and novice groups (*K* = 0.32 and 0.37, respectively). The IOR of the presence of solid components was moderate in the expert group (*K* = 0.52) and significantly higher than that in the other two groups (semi-experts, *K* = 0.09; and novices, *K* = 0.03).

Kim et al. reported the IOR of cyst communication with the duct as high (*K* = 0.69) and that of differentiating malignant from benign cysts as almost perfect (*K* = 0.92). The IOR of pancreatic cystic lesions is expected to be elevated with CH-EUS. Nonetheless, the challenge remains of eliminating the difference in IOR by years of experience with EUS.

## 5. Other Diseases

### 5.1. Lymphadenopathy

Differentiating benign from malignant lymphadenopathy is extremely important: EUS is appropriate for such evaluation [52,53,54]. Catalano et al. established the following EUS features as predictive of malignancy (in increasing order of importance): hypoechoic structures, sharply demarcated borders, rounded contours, and size >10 mm [52]. EUS-FNA is an efficient method for diagnosing lymphadenopathy, with recent reports describing sensitivity and specificity of 74–98% and 100%, respectively [55,56,57]. Although EUS-FNA has a high diagnostic value in lymphadenopathy, it is important to use only EUS to predict benign or malignant lesions.

Takasaki et al. evaluated the IOR of EUS features and proposed EUS diagnostic norms for lymphadenopathy based on the IOR [58]. The IOR was moderate for shape (*K* = 0.44) and fair for echogenicity, homogeneity, border, and hilum of the lymph node (*K* = 0.33, 0.34, 0.22, and 0.22, respectively). Moreover, they suggested the establishment of new EUS diagnostic criteria using shape, long axis (>20 mm), and short axis (>10 mm). Melo et al. studied the IOR of EUS diagnostic imaging for lymphadenopathy [59] and reported fair IOR for shape (*K* = 0.35) and moderate IOR for echogenicity and borders (*K* = 0.46 and 0.43, respectively). The overall IOR was high (*K* = 0.65).

Xia et al. evaluated the IOR of CH-EUS features for intra-abdominal lesions of undetermined origin. Results showed that about 80% of the eligible cases were in lymphadenopathy and that the IOR was almost perfect (*K* = 0.95). Furthermore, they reported that the sensitivity, specificity, positive predictive value, negative predictive value, and accuracy with which CH-EUS differentiated malignant from benign lesions were 96.3, 100, 100, 94.1 and 97.6% [60].

Several studies of lymphadenopathy using EUS-E have been conducted. Based on the general EUS-E patterns, lymph nodes were classified as benign (e.g., green color) or malignant (e.g., blue color) and scored in the study of Giovannini et al. [61]. Janssen et al. reported that the accuracy range among the examiners was 82–88% for benign lymph nodes and 85–87% for malignant ones. The IOR was almost perfect (*K* = 0.84) [62]. Based on results of a study by Larsen et al., the IOR of distinguishing benign or malignant lymphadenopathy was reported as moderate (*K* = 0.59) [63] (Table 4).

The IOR of lymphadenopathy is expected to be elevated through the use of CH-EUS and EUS-E. Nevertheless, further development of diagnostic criteria to predict benign or malignant lesions is warranted.

### 5.2. Gallbladder Wall Thickening

Gallbladder (GB) wall thickening might be caused by a broad spectrum of pathologies, including GB carcinoma, chronic cholecystitis, and adeno-myomatosis. Differentiation between GB carcinoma, adeno-myomatosis, and GB inflammatory diseases presenting as wall thickening is an important clinical issue because misinterpretation of GB wall thickening might result in unnecessarily extended surgery in patients with GB inflammatory diseases or delayed treatment in patients with GB carcinoma. Because it allows observation of the laminar structure of the GB wall, EUS is useful for the diagnosis of GB thickening [64,65]. Recently, some studies showed that CH-EUS might be useful for the diagnosis of GB disease [66,67]. Imazu et al. evaluated the IOR of diagnosing GB wall thickening using EUS and CH-EUS [68]. Univariate analysis results indicate that GB wall thickening of >12 mm and disruption of the GB wall layer structure on EUS and inhomogeneous distribution pattern of contrast agent on CH-EUS are significantly associated with malignant GB wall thickening with a high odds ratio. The addition of contrast enhancement to conventional EUS was extremely useful for differential diagnosis of GB wall thickening. Overall sensitivity and specificity for diagnosing malignant GB wall thickening of EUS and CH-EUS were 83.3 vs. 89.6, 65 vs. 98%, respectively. The IOR of the differential diagnosis of malignant or nonmalignant GB wall thickening was moderate with EUS (*K* = 0.51) and high with CH-EUS (*K* = 0.77) (Table 4).

The IOR of GB wall thickening diagnosis by CH-EUS is high; nevertheless, examination of more cases is necessary to further evaluate the usefulness of the method.

### 5.3. Subepithelial Lesion

EUS is also useful for diagnosing subepithelial lesions (SELs). The adjunctive application of CH-EUS can augment the diagnostic work-up of SEL. Recently, sensitivity, specificity, and accuracy rates of 100%, 63%, and 83%, respectively, were reported for the prediction of malignancy of gastrointestinal SEL by CH-EUS [69]. Gress et al. demonstrated that the IOR of SEL was almost perfect for cystic lesions (*K* = 0.80) and extrinsic compressions (*K* = 0.94), high for lipoma (*K* = 0.65), moderate for leiomyoma and vascular lesions (*K* = 0.53 and 0.54, respectively), and fair for other submucosal lesions (*K* = 0.34). Overall agreement among observers was strong (*K* = 0.63) [70]. Furthermore, significant association was found between the total years of experience with EUS and the number of correct answers (*p* = 0.01). Moreover, Fusaroli et al. [36] determined the IOR of the parameters (i.e., uptake, pattern, washout) examined in SEL of three types by CH-EUS. The IOR was large for uptake (*K* = 0.638), slight for pattern (*K* = 0.183), and fair for washout (*K* = 0.394) (Table 4). The IOR of SEL is high and independent of years of experience, suggesting that EUS is useful for diagnosing SEL.

### 5.4. Intrapancreatic Accessory Spleen

Accessory spleen (AS) might be encountered as an intrapancreatic lesion on EUS. It can present as a diagnostic dilemma because it might resemble other pancreatic pathologies, such as pancreatic adenocarcinoma and neuroendocrine tumors on imaging. Intrapancreatic AS (IPAS) is usually an incidental finding that requires no treatment [71,72]. Nevertheless, IPAS must be distinguished from other pancreatic neoplasms to avoid unnecessary interventions. Rodriguez et al. showed that an AS tends to be an isoechoic or hypoechoic mass with well-defined, smooth borders on EUS [73]. Kim et al. reported that the IOR of determining whether or not the pancreatic lesion is IPAS is fair (*K* = 0.37) [74] (Table 4). They also reported that the sensitivity and specificity for IPAS were greater than 70%.

Few studies have examined the IOR of AS; nonetheless, it has been suggested that combining EUS with other modalities might increase the diagnostic capabilities for AS.

### 5.5. Mucosa-Associated Lymphoid Tissue (MALT) Lymphoma

Several studies have demonstrated the reliability of EUS for staging MALT lymphoma before and after therapy [75,76,77,78]. Particularly, as reported earlier, EUS has sensitivity of 89%, specificity of 97%, and overall accuracy of 95% for depth of invasion [79]. Fusaroli et al. reported the IOR of EUS staging of gastric MALT lymphoma before and after treatment [80]. The overall IOR of T-stage was fair before and after treatment (*K* = 0.38 and 0.37, respectively). That of N-stage was large before treatment and fair after treatment (*K* = 0.63 and 0.34, respectively) (Table 4). Although the IOR of EUS staging of gastric MALT lymphoma varies, EUS for gastric MALT lymphoma might determine the treatment effects that were not observable using macroscopic morphology.

### 5.6. Gastrointestinal Malignancy 

EUS is used for staging of gastrointestinal malignancies. Yanai et al. reported the IOR of staging of submucosal invasion of superficial esophageal cancer (SEC) [81]. The IOR was fair (*K* = 0.46). It was suggested that EUS is useful for the evaluation of lymph node metastasis and might be useful in predicting the prognosis of SEC. Burtin et al. evaluated the IOR of staging of rectal cancer [82]. The IOR of uT1 tumors was fair (*K* = 0.40), uT2 tumors was slight (*K* = 0.20), and uT3 tumors was moderate (*K* = 0.58). In rectal cancer, EUS was also excellent for the evaluation of lymph node metastasis (*K* = 0.65). Roubein et al. was evaluated similarly for rectal cancer but stated only that the evaluation of lymph node metastases was a high IOR (*K* = 0.42) [83] (Table 4).

Reportedly, the IOR for staging of gastrointestinal malignancies cancers varies widely among observers. Further study of this point is required [84].

## 6. Future Perspectives

In recent years, as the development of artificial intelligence (AI) technology has progressed, it has been applied to the field of diagnosis by EUS [85,86,87]. Kuwahara et al. reported that the malignancy of IPMN can be diagnosed using AI with accuracy of 94%, which is higher than those obtained using conventional EUS features (40–60%) and endo-sonographer’s diagnosis (56%) [88]. Advances in AI are expected to be important for improving IOR-independent imaging capabilities in the field of EUS imaging.

## 7. Conclusions

EUS imaging has become a widely used approach for diagnosis of numerous diseases because of its high resolution and noninvasive nature. In addition, several EUS diagnostic criteria are extremely useful for diagnosing various diseases. However, the IOR of EUS diagnosis differs depending on the disease. Not only EUS findings with high IOR but also those with not necessarily high IOR are used as diagnostic criteria. Therefore, to further increase the value of EUS diagnosis, EUS diagnostic criteria with high diagnostic characteristics, based on EUS findings with high IOR, must be established.

## Figures and Tables

**Table 1:** Interobserver reliability of endoscopic ultrasonography (EUS) criteria in chronic pancreatitis.

EUS Findings	Topazian (2007) *n* = 20	Wallence (2001) *n* = 33	Stevens (2010) *n* = 50	Lieb (2011) *n* = 16	Gardner (2011) *n* = 44	Kalmin (2011) *n* = 36	Del Poza (2011) *n* = 69
Hyperechoic foci	0.12	0.29	0.21	0.19	0.39	NC	0.48
Strands	0.14	0.31	0.29	0.07	0.62	NC	0.55
Lobularity	0.23	0.51	0.16	0.53	0.44	NC	0.75
Cysts	0.48	0.32	0.35	NC	1	NC	0.66
Stones	0.03	0.38	0.36	0.78	NC	NC	NC
Duct dilation	NC	0.61	0.61	0.77	0.53	NC	0.75
Duct irregularity	0.2	0.29	0.5	0.60	NC	NC	NC
Hyperechoic MPD margin	0.2	0.36	0.33	0.34	0.53	NC	NC
Dilated side branch	0.09	0.18	0.46	0.11	NC	NC	NC
Rosemont classification	NC	NC	0.65	NC	NC	0.27	0.46

NC; not calculated.

**Table 2:** Interobserver reliability of contrast-enhanced harmonic endoscopic ultrasonography (CH-EUS) criteria in solid pancreatic lesion.

Author	Year	Disease	Degree of Experience	*K*-Value
Kitano et al. (*n* = 277)	2012	Adenocarcinoma	Experienced	0.56
Fusaroli et al. (*n* = 40)	2012	SPL	Overall Experienced Non-experienced	0.56 0.56 0.55
Gincul et al. (*n* = 100)	2014	SPL	Overall Experienced Non-experienced	0.66 0.76 0.65
Bunganic et al. (*n* = 116)	2018	SPL	Overall	0.60
Palazzo et al. (*n* = 81)	2018	P-NET	Overall Experienced Non-experienced	0.66 0.82 0.83

SPL, solid pancreatic lesion; P-NET, pancreatic neuroendocrine tumor.

**Table 3:** Interobserver reliability of EUS criteria for solid pancreatic lesion and pancreatic cystic lesion.

EUS Findings	Ahmad (2003) *n* = 31	Fusaroli (2012) *n* = 40	Jong (2011) By Expert *n* = 22	Jong (2011) By Semi-Expert *n* = 22	Jong (2011) By Novice *n* = 22
Contrast-enhanced	(-)	(+)	(−)	(−)	(−)
Solid component	0.43	NC	NC	NC	NC
Irregular pancreatic duct	0.29	NC	NC	NC	NC
Septation	0.30	NC	NC	NC	NC
Irregular margins	0.01	NC	0.65	0.32	0.37
Abnormal pancreatic parenchyma	0.01	NC	NC	NC	NC
Final diagnosis	0.24	0.58	0.43	0.09	0.30

NC, not calculated.

**Table 4:** Interobserver reliability of EUS criteria in other diseases.

Disease	Year	Author	*n*	Modality	*K*-Value
Lymphadenopathy	2019	Takasaki et al.	68	EUS	0.65
	2010	Xia et al.	43	CH-EUS	0.95
	2007	Jassen et al.	50	EUS-E	0.84
	2011	Larsen et al.	61	EUS-E	0.59
Gallbladder Wall Thickening	2014	Imazu et al.	36	EUS	0.51
	-	-	-	CH-EUS	0.77
Subepithelial Lesion	2001	Gress et al.	20	CH-EUS	0.63
	2012	Fusaroli et al.	40	CH-EUS	uptake 0.64, pattern 0.18 washout 0.39
Intrapancreatic Accessory	2019	Kim et al.	12	EUS	0.37
MALT Lymphoma	2002	Fusaroli et al.	54	EUS	T-stage before and after treatment = 0.38 and 0.37 N-stage before treatment and fair after treatment = 0.63 and 0.34
Staging of superficial esophageal cancer	2003	Yanai et al.	31	EUS	0.31
Staging of rectal cancer	1997	Burtin et al.	27	EUS	T2 0.20 T3 0.58

MALT, Mucosa-associated Lymphoid Tissue.

## Supplementary Materials

The following are available online at https://www.mdpi.com/2075-4418/10/11/953/s1: Table S1: Endoscopic ultrasound criteria for the diagnosis of chronic pancreatitis, Table S2: Classification of patients based on endoscopic criteria.
