# Supplementary material for: Interobserver Reliability of Endoscopic Ultrasonography: Literature Review
Source: Diagnostics (Basel). 2020 Nov 15;10(11):953. doi: 10.3390/diagnostics10110953 (PMC7696989; doi:10.3390/diagnostics10110953)
Supplement: Supplementary file 1 [file diagnostics-10-00953-s001.pdf]

Supplementary Table.1 Endoscopic ultrasound criteria for the diagnosis of chronic pancreatitis.

Standard criteria	Rosemont criteria
**Parenchymal criteria**	**Major criteria A**
Hyperechoic foci	Hyperechoic foci (>2 mm in length/width with shadowing)
Hyperechoic strands	Major duct calculi (echogenic structure[s] within the MPD with acoustic shadowing)
Hypoechoic lobules, foci or areas	**Major criteria B**
Cyst	Lobularity (≥3 contiguous lobules = 'honeycombing')
**Duct criteria**	**Minor criteria**
Irregular duct contour	Cyst (anechoic, round/elliptical with or without septations)
Visible side branches	Dilated duct (≥3.5 mm in body or >1.5 mm in tail)
Hyperechoic duct margin	Irregular duct contour (uneven or irregular outline and ectatic course)
Dilated main duct	Dilated side branch (>3 tubular anechoic structures each measuring ≥1 mm in width, budding from the MPD)
Stone	Hyperechoic duct wall (echogenic, distinct structure>50% of entire MPD in the body and tail)
	Hyperechoic strands (≥3 mm in at least 2 different directions with respect to the imaged plane)
	Hyperechoic foci (>2 mm in length/width that are nonshadowing)
	Lobularity (>5 mm, noncontiguous lobules)

Supplementary Table. 2 Classification of patients based on endoscopic criteria.

Standard criteria	Rosemont criteria
**Normal (or low probability)**	**Consistent with**
0–2 criteria	2 major A
**Indeterminate or intermediate probability**	1 major A + 1 major B
3–4 criteria	1 major A + ≥3 minor
**High probability**	**Suggestive of**
5–9 criteria	Major A + <3 minor
	Major B + ≥3 minor
	≥5 minor, no major
	**Indeterminate**
	Major B alone + <3 minor
	**Normal**
	<3 minor, no major
